# Oscillatory But Not Aperiodic Frontal Brain Activity Predicts the Development of Executive Control From Infancy to Toddlerhood

**DOI:** 10.1111/desc.13613

**Published:** 2025-02-09

**Authors:** Josué Rico‐Picó, M. del Carmen Garcia‐de‐Soria Bazan, Ángela Conejero, Sebastián Moyano, Ángela Hoyo, María de los Ángeles Ballesteros‐Duperón, Karla Holmboe, M. Rosario Rueda

**Affiliations:** ^1^ Department of Experimental Psychology University of Granada Granada Spain; ^2^ Mind, Brain and Behavior Research Center (CIMCYC) University of Granada Granada Spain; ^3^ Department of Developmental Psychology University of Granada Granada Spain; ^4^ Department of Psychobiology University of Granada Granada Spain; ^5^ School of Psychological Science University of Bristol Bristol UK

**Keywords:** attention, EEG, executive control, infancy, brain oscillatory activity, ECITT

## Abstract

Executive control (EC) emerges in the first year of life, with the ability to inhibit prepotent responses (inhibitory control [IC]) and to flexibly readapt (cognitive flexibility [CF]) steadily improving. Simultaneously, electrophysiological brain activity undergoes profound reconfiguration, which has been linked to individual variability in EC. However, most studies exploring this relationship have used relative/absolute power and tasks that combine different executive processes. In addition, brain activity conflates aperiodic and oscillatory activity, which hinders the interpretation of the relationship between power and cognition. In the current study, we used the *Early Childhood Inhibitory Touchscreen Task* (ECITT) to examine the development of EC skills from 9 to 16 months in a longitudinal sample, and related performance of the task to resting‐state EEG (rs‐EEG) power, separating oscillatory and aperiodic activity. Our results showed improvement in IC but not in CF with age. In addition, alpha and theta oscillatory activity were concurrent (9‐mo.) and longitudinal predictors of CF in toddlerhood, whereas the aperiodic exponent of the EEG signal did not contribute to EC. These findings demonstrate the relevance of oscillatory brain activity for cognitive development and provide an early brain marker for the early development of EC.

## 1 Introduction

One of the most notable human capacities is the ability to consciously adjust behavior to achieve goals. Executive control (EC) refers to a form of action selection and monitoring that involves attention‐based goal‐directed bias over habitual responses (Botvinick et al. 2001; Rueda, Moyano, and Rico‐Picó 2021). When the context is stable it is very useful to develop response tendencies that minimize the cognitive resources needed for response selection. However, if conditions change, attention is necessary to detect the change, inhibit the action tendency, and adjust the response to the new situation. Thus, attention, inhibitory control (IC), and switching are key EC processes. Importantly, EC is central to cognitive and socio‐emotional adjustment, and difficulties with these functions have been associated with poorer outcomes in diverse life domains, such as academic performance along development (Rueda, Checa, and Rothbart 2010), and wealth and health levels in adulthood (Moffitt et al. 2011).

**Summary**

- Inhibitory control improves from infancy and toddlerhood evaluated with a protocol that isolates it from memory processes.
- Theta peak frequency was a positive concurrent predictor of cognitive flexibility in infants.
- Larger alpha and theta oscillatory power at 9 months of age positively predicted cognitive flexibility at 16 months of age.

**Declaration of AI‐Assisted Technologies in the Writing Process**

During the preparation of this study, the authors used *Paperpal* to review the grammar and consistency of the text. After using this tool, the authors reviewed and edited the content as required and took full responsibility for the publication.

Previous research suggests that the developmental foundation of EC occurs during the first year of life (Diamond 2013; Fiske and Holmboe 2019; Holmboe et al., 2018; Hendry et al. 2016). However, some of the tasks employed to measure IC in infants and toddlers present some limitations (Holmboe et al. 2021). Classic EC paradigms, such as the Go/No‐Go task, require understanding verbal instructions and/or rely on several cognitive processes (Conejero and Rueda 2017; Holmboe et al. 2021). For instance, widespread research based on the A‐not‐B task suggests an improvement in IC capacity in the first 2 years of life (Clearfield et al. 2006; Diamond 1990, 1985; Holmboe et al. 2018; Johansson, Forssman, and Bohlin 2014); however, this improvement in the performance may be driven by age‐related changes in working memory (WM) capacity given its necessity of retaining the location and implement it to current behavior (Holmboe 2021).

To overcome these limitations, Holmboe et al. (2021) recently developed the “*Early Childhood Inhibitory Touchscreen Task”* (ECITT). This task was inspired by the rationale behind the A‐not‐B and Go/No‐Go paradigms. In this task, infants are taught to press a target button on a tablet screen to obtain a positive reward. The target button appears most of the time on one side of the screen (prepotent location) and occasionally on the other side (inhibitory location). Thus, infants must override their tendency to touch the prepotent side when the target appears on the less frequent side (IC). In addition, cognitive flexibility (CF) can be measured by assessing the impact of switching the location of the target with respect to the previous trial. Besides, the ECITT does not require verbal instructions or memorization of the location, thus providing a clean measure of EC processes based on a response‐reward contingency rationale. There is evidence that infants as young as 10 months can perform this task, showing a significant improvement of EC in the transition between infancy and toddlerhood (Hendry et al. 2022; Holmboe et al. 2021). In the original study presenting the ECITT task, 3‐year‐old children demonstrated superior accuracy in inhibitory trials compared to a 2‐year‐old group. Furthermore, this improvement was also observed between 18‐ and 21‐month‐old children relative to 2‐year‐old subjects (Holmboe et al. 2021). In a subsequent study, Hendry and colleagues (2022) identified an enhancement in switching capacity between 10 and 16 months of age, although the age‐related change was not statistically significant in the IC composite score.

The improvement in EC occurs alongside the maturation of the frontal brain regions (Bell and Cuevas 2012; Cuevas and Bell 2022; Diamond 2013; Fiske and Holmboe 2019). To study this relationship, most experiments have employed EEG recordings at rest (rs‐EEG) because of their adaptability and ease of use in infants (Saby and Marshall 2012). The gold standard measure of rs‐EEG in developmental samples is the absolute or relative power of canonical bands such as theta (3–6 Hz) and alpha (6–9 Hz). Longitudinal studies have shown a profound reconfiguration of these measurements in the first years of life (e.g., an increase in alpha power), highlighting the potential of EEG to capture brain maturation (Anderson and Perone 2018; Stroganova, Orekhova, and Posikera 1999). More importantly, individual differences in relative power have been associated with EC development during infancy (Bell and Cuevas 2016). For example, larger frontal alpha power at rest is related to better attention and EC skills (Bell 2001; Bell and Fox 1997, 1992; Whedon, Perry, and Bell 2020; Wolfe and Bell 2004). In addition, infants’ theta modulation at rest or its values in evoked paradigms suggests that it is related to high‐order cognition (Begus, Southgate, and Gliga 2015, Braithwaite et al. 2020; Conejero et al. 2016).

One limitation of using absolute/relative power is that it conflates narrow and broadband activity (Donoghue et al. 2020; Ostlund et al. 2022). When the EEG signal is analyzed in terms of power, most of the energy follows a power‐law decaying curve (aperiodic), whereas only the peaks of energy above this curve represent oscillatory activity (Voytek and Knight 2015). When these components have been isolated, aperiodic activity is the most important predictor of several band ratios (e.g., theta/beta), except for the alpha band (Donoghue, Dominguez, and Voytek 2020; Rico‐Picó et al. 2023). In addition, the aperiodic curve flattens throughout the lifespan (Cellier et al. 2021; McSweeney et al. 2021; Rico‐Picó et al. 2023; Schaworonkow and Voytek 2021), and only the alpha band exhibits the same trajectory of oscillatory (vs. relative) power (Rico‐Picó et al. 2023; Schaworonkow and Voytek 2021). Thus, previous results may have been misled by co‐occurring aperiodic maturation, particularly in the theta band, which is influenced by the aperiodic exponent (Donoghue et al. 2020). Furthermore, recent studies have linked disorders that compromise EC to the aperiodic components of the EEG signal highlighting its relevance in cognitive processes (Karalunas et al. 2021; Shuffrey et al. 2022).

In the case of the ECITT task, a recent fNIRS (functional near‐infrared spectroscopy) study corroborated the involvement of frontal areas by recording brain activity while children performed a blocked version of this task (Fiske et al. 2022). In this version, the control (only prepotent trials) and experimental (50% of inhibitory trials) blocks were alternately presented. In the original study, 10‐months infants exhibited increased brain activity in frontal areas during the experimental blocks (Fiske et al. 2022), which occurred in more widespread brain areas at 16‐months of age (Fiske et al. 2024). However, how fine‐grained ECITT indices are related to frontal rs‐EEG has not been addressed, particularly focusing on the distinction between aperiodic and oscillatory components. Our study aimed to fill this gap in the literature, and our goals were twofold: (1) to investigate the early development of EC from 9 to 18 months of age and (2) to examine the contribution of aperiodic and oscillatory power to individual differences in the ECITT task performance and development over that age range.

Given previous results with the ECITT (Hendry et al. 2022) and the A‐not‐B task (e.g., Clearfield et al. 2006), we predicted a general improvement in infant performance on the ECITT task during the transition from infancy to toddlerhood. In addition, we predicted that a flatter aperiodic exponent and higher alpha power would be significantly related to EC development. However, as theta power ratios are highly related to aperiodic activity (Rico‐Picó et al. 2023), we anticipated that the association between theta power and EC might disappear when controlling for the aperiodic exponent.

## 2 Method

### 2.1 Participants

Families were recruited from hospitals and nurseries at the Virgen de las Nieves Hospital and in the metropolitan area of Granada City (Spain) via informative rounds conducted by the study researchers and advertising. If families showed a willingness to be further contacted, parents/legal guardians were called when the babies were 6 months old to schedule a session. Infants were followed up at 9 and 16–18 months. The current study only included data from the second and third follow‐up sessions, as ECITT was not administered at 6 months of age. Prematurely and/or low weight at birth infants (<37 weeks of gestation; <2.5 kg of weight), and those at risk for neurodevelopmental disorders were excluded from the final sample (*n* = 18; Figure S1). All families received vouchers from a local toy store to compensate for their participation in each session.

Eighty‐seven 9‐month‐old infants participated in the 9‐month‐old session, of which 24 were excluded due to insufficient valid data (see Section 2.2.1.3) and three due to technical issues (valid *n* = 60, 68.97% retention rate). In session 2, at 16–18 months of age, 75 children completed the ECITT task, of which seven were excluded because they had insufficient valid data and three due to technical difficulties (*n* = 65, 86.67% retention rate). This exclusion rate aligns with previous ECITT studies (e.g., Hendry et al. 2022 excluded approximately 22% of 10‐month‐old infants). Infants were required to have valid data in at least one session and were permitted to have missing data in the other session to be included in the analysis. Seventy‐four infants met this criterion: 34 had valid data in both sessions, while 23 and 17 had valid data only at the 9‐month and 16‐month sessions, respectively (Table 1 and Figure S1).

**TABLE 1:** Demographic information about the sample included in the behavioral analysis.

Session	Sex	*N*	Session age (days)	Income to needs
9‐mo.	F	26	284.65 (9.41)	1.36 (0.65)
	M	31	285.22 (7.66)	1.4 (0.77)
16‐mo.	F	25	514.79 (22.89)	1.36 (0.65)
	M	26	522.75 (25.82)	1.4 (0.77)

*Note*: This table presents information of infants who had valid data and were included in the linear mixed models exploring the development of EC with the ECITT task. Given that we estimated missing data, the number of infants included in the models was 74. This table shows the *M* (SD).

To investigate the relationship between EEG and the ECITT task, valid data from both protocols were required. This analysis examined the concurrent relationship between brain function and behavioral performance (e.g., both EEG and ECITT at 9), as well as whether EEG at 9 months could predict ECITT performance at 16 months (longitudinal analysis). From the 60 infants with valid ECITT data at 9 months, 17 declined to wear the net, and 6 were excluded due to insufficient epochs (see Section 2.2.2.2), resulting in 35 participants included in the final analysis. At 16 months of age session, 35 infants had both valid data in the ECITT task and the EEG (27 infants declined to wear the net, and one infant did not meet the minimum number of epochs required). Finally, for the longitudinal analysis, 35 infants out of 65 had both valid EEG data in the EEG at 9 months and ECITT at 16 months of age (excluded due to lack of EEG *n* = 28, excluded to insufficient EEG clean epochs *n* = 2). The demographic details of the final sample are presented in Table 2. The exclusion rate aligns with previous EEG studies, where approximately 50% of participants are discarded when combining behavior and neuroimaging in very young populations (e.g., Braithwaite et al. 2020).

**TABLE 2:** Descriptive statistics of the sample included in the linear regression models predicting ECITT performance based on rs‐EEG.

		*n* (female)	Session age	Valid trials	*rs‐EEG*	
					*Clean epochs*	*R^2^*
Concurrent	*9‐mo*.	37 (15)	284.67 (10.16)	18.84 (3.27)	15.40 (9.26)	0.98 (0.01)
	*16‐mo*.	37 (21)	513.40 (20.51)	29.63 (2.53)	15.35 (9.51)	0.99 (0.01)
Longitudinal	—	35 (18)	*9‐mo*.: 283.66 (10.68)	*16‐mo*.: 28.07 (4.92)	*9‐mo*.: 12.69 (6.98)	*9‐mo*.: 98 (0.01)
			*16‐mo*.: 515.25 (21.78)			

*Note*: This table presents the data of infants who had valid data for both the rs‐EEG and ECITT tasks. Being included in the behavioral analysis was not a requirement for inclusion in the relational analysis. Thus, the sample varied slightly between the participants in both analyses. Session age (days). The valid trials corresponded to the ECITT task. *R^2^* represents the fit of power spectrum decomposition. This table presents the information for when the EEG and ECITT were collected in the same wave (concurrent) and in the longitudinal analysis predicting ECITT performance at 16‐months. based on EEG data at 9‐months. Data are presented as M (SD).

Abbreviations: rs‐EEG = resting‐state EEG.

Participants' demographic information was informed by parents via an online questionnaire that was sent following the first session. Families were asked about ethnicity, language spoken at home, income, education, and occupation. In this study, all infants were Hispanic, white, and monolingual. Seventy‐three percent of families reported an overall household income above the poverty level according to the Spanish National Institute of Statistics (*M* = 1.38 times the poverty line income). Most parents possessed either a bachelor's degree or postgraduate education (mothers: 71.18%, fathers: 53.02%), while the rest had achieved at least secondary education, except for one father who had not completed basic schooling. With respect to occupation, 32.35% of mothers and 7.35% of fathers were unemployed, with the majority employed in administration, teaching, and management (see Supporting Material S1. Demographic description of the sample for more details).

### 2.2 General Procedure

The research presented in this paper is part of a larger longitudinal study that encompasses a wider set of tasks than those presented here. Before performing the ECITT task in both the 9‐ and 16‐month sessions, participants conducted different eye‐tracking protocols that took about 10–15 min, and only in the 16‐month‐old session the ECITT was followed by a spatial reasoning task. The rs‐EEG protocol was always conducted at the end of the session immediately after the participants were presented with an event‐related protocol lasting approximately 7 min. In all procedures, the tasks were video recorded, and the infants were seated on their caregiver's lap. Parents were instructed to remain silent and not interact with their children during the task.

#### 2.2.1 ECITT

##### 2.2.1.1 Apparatus

The stimuli were presented on an Apple iPad tablet (screen:9.7 inches; 2048 × 1536 pixels). The software of the ECITT was programmed by Henrik Dvergsdal (for details, see Holmboe et al. 2021) and it is available online (see: https://ecitt.app).

##### 2.2.1.2 Protocol

**FIGURE 1:**
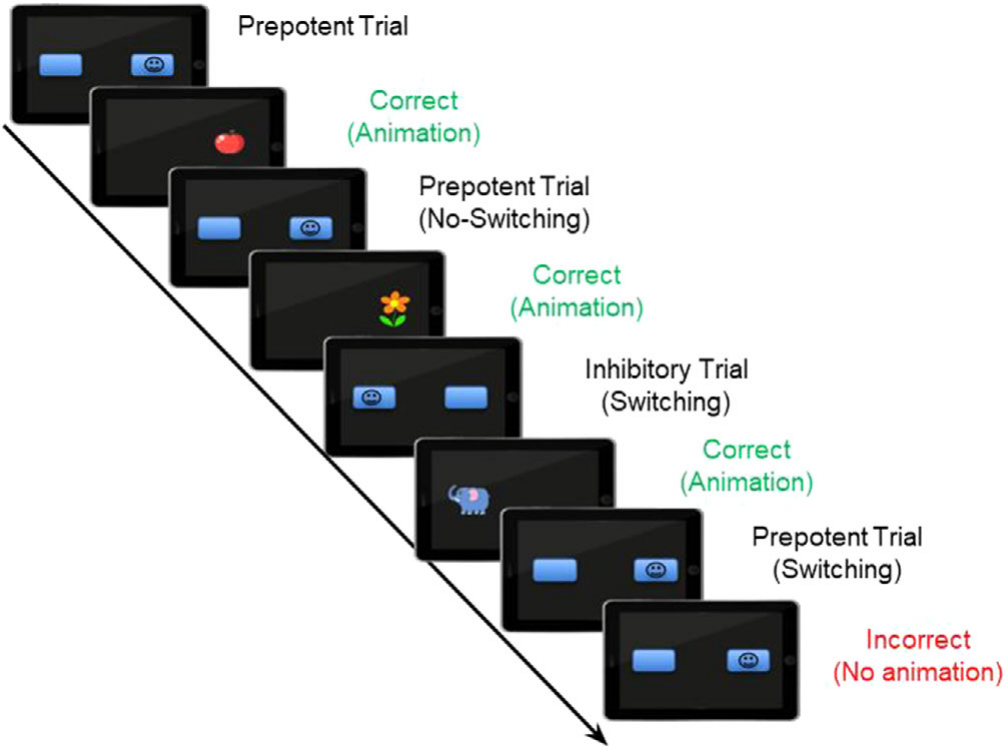
Schematic representation of the experimental trial sequence in the ECITT task. ECITT = Early Childhood Inhibitory Touchscreen Task.

We followed the standard ECITT protocol described by Hendry et al. (2022). The session started with a *Familiarization Phase* to allow them to interact with the tablet. In this phase, the experimenter encouraged the infants to touch the butterfly displayed on the tablet screen. If the child did not touch it, the experimenter modeled the action and provided positive feedback when the child imitated her/him. Afterward, the infants completed a *Practice Block* in which a centrally positioned blue button with a “smiley face” (target button) appeared on the screen, and the experimenter prompted the infant to touch it. A short animation with music was presented as positive feedback after the infant tapped the button. This step was used to create an association between the target and the positive feedback. Once the infant demonstrated competence in touching the button, the *Experimental Task* was initiated. During the experimental trials (Figure 1), two blue buttons, one empty (blank button), and one with the “smile face” on it, were displayed on the sides (right or left) of the screen. The experimenter gently encouraged the infant to touch the target by saying ‘Can you touch the smiley face?’ Correct touches were immediately followed by child‐friendly feedback (a short cartoon animation with music), whereas the stimuli remained unchanged following incorrect, or off‐button touches.

##### 2.2.1.3 ECITT Variables

Each infant performed a single block of 32 experimental trials. The target button appeared more frequently on one side of the screen (75% prepotent location) and less frequently on the opposite side (25% inhibitory location). The prepotent location was counterbalanced between participants but was the same for each infant in the two longitudinal sessions (at 9 and 16 months of age). The experimental block began with at least three consecutive prepotent trials to establish an initial tendency. The trial selection was then pseudo‐randomized, allowing a maximum of five prepotent trials in a row and a maximum of two consecutive inhibitory trials.

Infant behavior was video recorded and subsequently reviewed by a trained researcher. In this post‐hoc evaluation of the task, the researchers excluded invalid trials and determined the trial validity and accuracy. Trials with at least one of the following conditions were marked as invalid: (a) the infant responded without visual focus on the device, (b) the infant touched the screen with both hands, (c) reaction time was below 300 ms, (d) there was parental indication of the correct answer. To assess the reliability of the post‐hoc codification, another reviewer codified a subset of the videos (*N* = 50) at ages 9 and 16 months for both accuracy and validity. Then, we calculated Cohen's Kappa reliability (R, *psych* package). This analysis demonstrated good intercoder reliability at 9‐month‐old (*n* trials = 1373, *k* accuracy = 0.85, *k* validity = 0.80) and 16‐month‐old (*n* trials = 1515, *k* accuracy = 0.90, *k* validity = 0.84) sessions. Based on the codified values, infants were excluded if they did not reach 60% accuracy in prepotent trials, had fewer than two inhibitory trials, or did not complete at least 50% of the trials. Our final sample for behavioral analysis included infants with valid data in the two sessions or who had one session with valid data, and the other was missing (9‐months session: *M* valid trials = 18.74; SD valid trials = 3.61; 16‐months session: *M* valid trials = 28.59; SD valid trials = 4.3; Table 1 and Supporting Material for further details of the socioeconomic status of the sample).

Similar to previous studies with very young participants (e.g., Hendry et al. 2022), we did not consider infants’ RT given that babies often behave erratically between trials while performing the task. Thus, task performance was measured as the percentage of correct valid answers under three conditions: prepotent non‐switch (PNS), prepotent switch (PS), and inhibitory switch (IS). The PNS trials were those in which the target appeared at a prepotent location, as in the previous trial. In the PS trials, the target appeared at the prepotent location but followed a presentation in the inhibitory location. In the IS trials, the target appeared at the inhibitory location following the presentation at the prepotent location. Unlike previous studies using the ECITT, we decided to consider sequential changes in the target location to disentangle IC from response‐switching costs, an effect related to CF and control (Koch, Frings, and Schuch 2018). We also computed two general indices of performance in the task: the Inhibitory Effect (PS Accuracy—IS Accuracy) and the Switching Effect (PNS Accuracy—PS Accuracy). The Inhibitory Effect measures the cost of accuracy owing to failures to inhibit touching the prepotent location, whereas the Switching Effect reflects the general costs of changing from one location to another. Note that the performance indices in our study vary from those used in previous studies on the ECITT (e.g., Hendry et al. 2022). We provide the behavioral results using these indices in the Supporting Results section.

#### 2.2.2 Electroencephalography Recording at Rest

##### 2.2.2.1 Apparatus and Protocol

We recorded EEG activity at rest in both 9‐ and 16‐month‐old sessions. The resting protocol consisted of 4 min of recording, divided into two blocks. In the first block, the experimenter blew soap bubbles in front of the infants. In the second block, a video featuring geometrical shapes and music was presented on a computer screen. This approach was implemented to increase the likelihood of obtaining a valid EEG record by extending the overall duration of the protocol and mitigating boredom and fussiness in infants. In 9‐month‐old session, 31.82% (SD = 33.73%) of epochs corresponded to the video block, while the percentage in 16‐month‐old session was 36.87% (SD = 32.87%). Notice that the oscillatory and aperiodic parameters of the EEG power were correlated at both ages (9‐mo.: *n* = 36, all rs > 0.42, all *p*s_‐FDR‐corrected_ < 0.024; 16‐mo.: *n* = 29, all rs > 0.38, all *p*s_‐FDR‐corrected_ < 0.044) indicating that the brain activity registered under the resting protocol is similar in both blocks.

The EEG signal was recorded with a 128‐channel geodesic net (EGI Geodesic Sensor Net, Eugene, OR, USA) using the EGI software Net Station 4.3 with a digitalization rate of 1000 Hz. The online signal was recorded with reference to the Cz electrode and filtered with elliptical low‐pass (100 Hz) and high‐pass (0.1 Hz) hardware filters. The session was videotaped, and an experimenter coded the infants’ behavior to discard moments with parental interruptions and/or the baby's fussiness.

##### 2.2.2.2 EEG Processing

**FIGURE 2:**
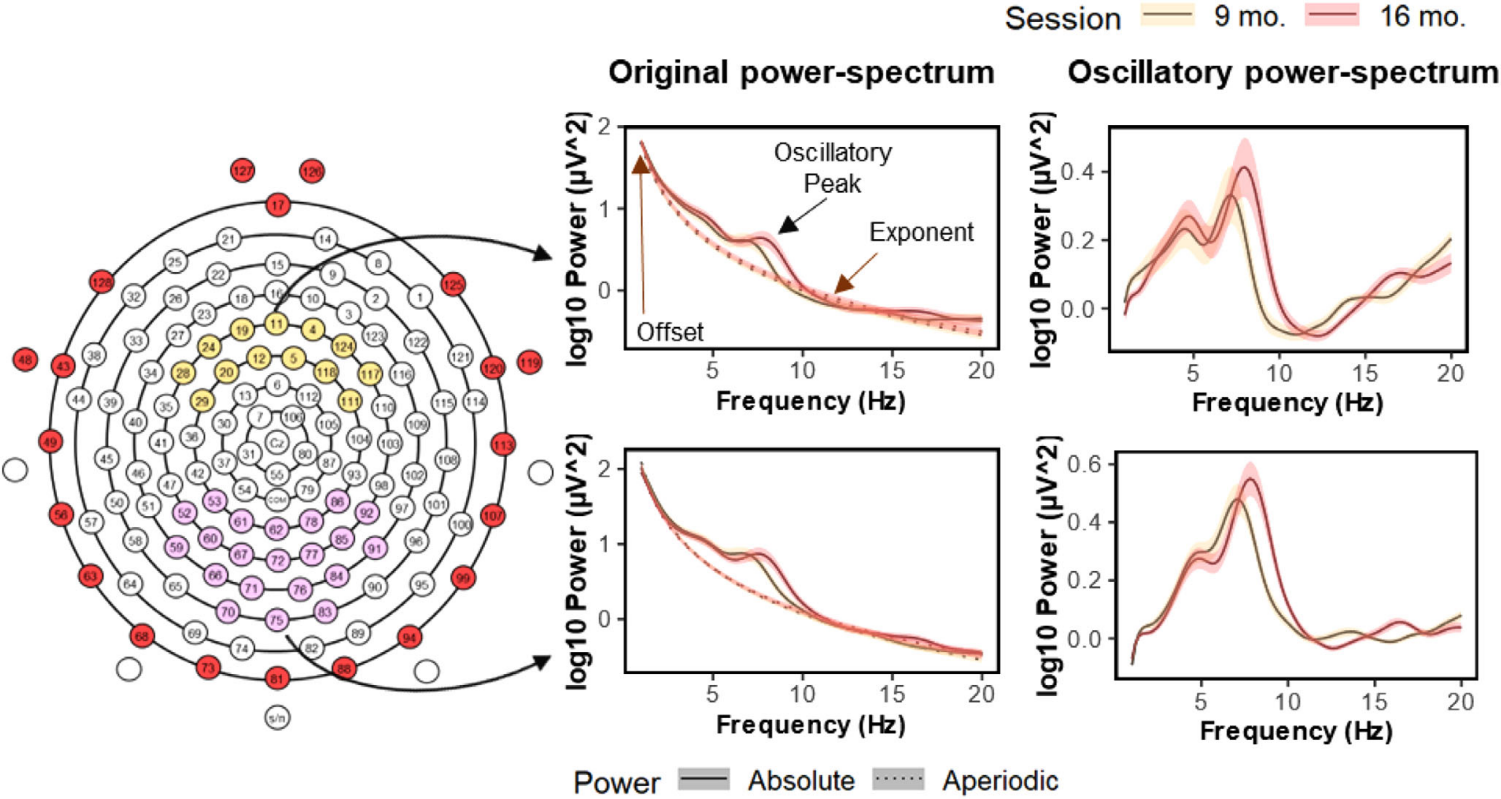
The EEG layout employed in this study (left) and power spectrum fit the Specparam toolbox. The first column represents the absolute power and aperiodic background curve, whereas the second column displays the oscillatory power spectrum after subtracting the aperiodic activity. *Note*: Layout colors represent excluded electrodes (red), occipital‐parietal (pink), and frontal (yellow) clusters. The lines in the graphs represent the mean and the shaded lines correspond to twice the standard error.

To process the EEG signal, we followed the preprocessing steps presented by Rico‐Picó et al. (2023), which combined the MADE (Debnath et al. 2020) and APICE (Fló et al. 2022) pipelines in EEGlab. The signal was filtered (FIR; 0.2–48 Hz), and boundary electrodes (*n* = 20) were excluded from further processing because they were excessively noisy (Figure 2). Then, global bad channels were detected using the EEGlab plug‐in FASTER (Nolan, Whelan, and Reilly 2010) and removed from the dataset. Next, we created a copy of the dataset and computed an independent component analysis (ICA; high pass filtered to 1 Hz; segmented into 1s epochs; threshold = ±1000 µV; detect activity between 20 and 30 Hz). Bad components were detected with the adjusted ADJUST (Leach et al. 2020), which were copied into the original dataset and removed from the recording. Over the continuum, we detected the “transient bad moments” with an adaptive threshold based on the power spectrum, amplitude, and variance of the signal. These moments were targeted using principal component analysis (PCA); if they lasted less than 100 ms, the pipeline removed the components containing up to 0.90 of the variances. Then, the signal was divided into 5 s epochs (50% overlapping), and we redefined the “transient bad moments.” If more than 30% of the signal was noisy within one epoch, it was removed. Otherwise, the channels of bad moments were interpolated within the epoch. Finally, the global bad channels excluded by FASTER were reintroduced using spherical interpolation, and the signal was re‐referenced to the average. Over the remaining epochs, we applied a ±110 µV voltage threshold, and in the case of 20% of the channels surpassing it, the epoch was discarded. Finally, the experimenter visually inspected the segments and removed the bad segments if necessary.

The inclusion threshold based on the number of epochs was determined by the reliability of the oscillatory and aperiodic parameters (see Supporting Material). To this end, we calculated Spearman‐Brown split‐half reliability from 2 to 20 epochs in 1 epoch increments across 5000 iterations (see Troller‐Renfree et al. 2021). This procedure was conducted for each variable in its corresponding electrode region and separately for each session. Generally, theta peak frequency demonstrated the lowest reliability, while the aperiodic components exhibited the highest. Furthermore, this analysis indicated that 25s of data was sufficient to yield excellent reliability values (*r*_sb_ > 0.8), except for alpha and theta peak frequency, which attained only a substantial reliability value (*r*_sb_ > 0.7). Thus, we excluded infants with less than 25s (i.e., 5 epochs) of valid data (concurrent analysis: 9‐month‐old *n* = 6, 16‐month‐old. *n* = 1; cross‐sessions analysis: *n* = 2), missing data in the EEG (vs. ECITT task; concurrent analysis: 9‐month‐old *n* = 17, 16‐mo. *n* = 27; cross‐sessions analysis: *n* = 28), or with more than 10 global electrodes detected by FASTER (*n* = 0). Females had more epochs before the preprocessing at 9‐months than males (all *t*s > 16, all *p*s < 0.001), while males had longer recordings at 16‐months (*t* = 25, *p* < 0.001). However, groups did not differ in cleaned epoch number according to either the age (all *t*s < 1.05) or sex (*t* = 1.10) variables (see Table 2 for further details).

##### 2.2.2.3 EEG Power Computation

We obtained the aperiodic and oscillatory parameters using the *Specparam* toolbox (Donoghue, Haller et al. 2020; https://pypi.org/project/specparam/) through a MATLAB wrapper (https://github.com/bfbarry/EEGLAB‐specparam). This toolbox models the results of an FFT (1–20 Hz) provided by the *pop_spectopo* function into aperiodic and oscillatory power. It considers the power at each frequency (P(*f*)) as a combination of aperiodic (L(*f*)) and oscillatory (G; Σn Gn (*f*)) activities. The aperiodic curve is defined as L(*f*) = 𝑏—log[fx] where 𝑏 is a constant offset and 𝜒 is the exponent of the decaying curve, while the oscillatory (G) peaks are modeled as Gaussian curves. The *specparam* fitting parameters were similar to the ones employed in previous infants studies (peak width limits: [2.5–12 Hz]; maximum number of peaks: 5; aperiodic mode: fixed; peak threshold: 2; see Rico‐Picó et al. 2023; Schaworonkow and Voytek 2021). To ensure reliability of the results, we excluded channels with fit values below *R* ^2^ = 0.95. Infants had to have at least 50% of electrodes surpassing that threshold per ROI to be included in the analysis (excluded *n* = 0; electrodes excluded *M*_9‐mo._ = 4.2%, *M*_16‐mo._ = 2.1%; Table S2; Figure S3). In the ROIs included in the analysis (see Figure 2), the parieto‐occipital clusters had a better fit than the frontal area (all *t*s > 7.04, all *p*s < 0.001) but without differences between parietal and occipital areas (*t* < 1). At 9 months of age, the goodness of fit was lower than in 16‐month‐old session (all *t*s > 4.17, all *p*s < 001), and females had larger fit values than males independent of session (all *t*s > 5.2, all *p*s < 001).

We computed the oscillatory power in two frequency bands: theta and alpha. Alpha and theta frequency ranges were centered on the infants’ individual alpha (IAF) and theta (ITF) peak frequencies over the parieto‐occipital (Figure 2) clusters. We selected this ROI and adapted the frequency ranges because the parieto‐occipital area shows larger reconfiguration in infants when exposed to resting conditions and the peak frequency steadily increases in the first years of life (Marshall, Bar‐Haim, and Fox 2002; Freschl et al. 2022; Rico‐Picó et al. 2023; Stroganova, Orekhova, and Posikera 1999). Given the age of the infants included in the study, we determined that an oscillatory peak provided by *specparam* toolbox was an alpha peak if it was over the 6–9 Hz interval, while we considered 2.5–5.5 Hz range to determine if a participant had a theta peak. After obtaining the IAF and ITF, we constructed a frequency range considering the bandwidth for alpha and theta peaks by (e.g., IAF ± (Band‐Width_alpha_/2)). To obtain the oscillatory power, we subtracted the aperiodic background curve from the absolute power for each frequency band. We also considered the aperiodic exponent, IAF, and ITF in the analysis. Oscillatory power and exponent were computed over the frontal ROI, whereas the IAF and ITF were extracted from the parieto‐occipital cluster. Each measurement was calculated per electrode and then averaged over the cluster, excluding the electrodes with goodness of fit *R* ^2^ < 0.95.

### 2.3 Analysis Plan

#### 2.3.1 Behavioral Change and Stability

We used linear mixed models to analyze age‐related changes in ECITT performance. The models for the accuracy values of PNS, PS, and IS included Age, Type of Trial, and their interaction as fixed effects in a single model, whereas the models of the composite indices were computed independently for each variable. We tested two random structures in the models (intercept vs. intercept + slope per participant). When a random slope was introduced, the resulting models were singular. Therefore, all reported analyses included only random intercepts per participant. We employed the approximations of Satterthwaite and Nakagawa's et al. (Nakagawa, Johnson, and Schielzeth 2017) *R^2^* to compute the degrees of freedom and the effect size of the model, respectively. When the residuals were non‐normally distributed, we employed Tucker ladder transformation and used the model in the first step. To compute individuals’ longitudinal stability in the performance of the task, we conducted linear regressions including 9‐month‐old performance as a predictor. We accounted for the missing values with maximum likelihood estimation to minimize the loss of statistical power in the following cases: (1) the participant experienced technical problems, and we could not conduct the ECITT task; (2) the participant did not attend one of the experimental sessions (Enders 2013; Graham 2009; Matta, Flournoy, and Byrne 2018). Little's MCAR test determined that missing data were missing completely at random (X^2^ = 0.69, *p* = 0.708). Additional analyses also showed that the socioeconomic status of the family did not vary among infants with and without complete data (9‐month‐old: *t (*58) = 1.1, *p* = 0.136; 16‐month‐old: *t* (58) = −0.603, *p* = 0.274).

#### 2.3.2 EEG and ECITT Relationship

The R package *glmulti* (Calcagno and Mazancourt 2010) was used to study the relationship between ECITT performance and functional brain power. This package conducts linear regressions by creating all the possible combinations given the matrix of independent variables and finds the one that fits the best to predict the dependent variable. The model selection in this package is based on AICc, thus correcting the number of independent variables. We examined concurrent (e.g., 9‐months EEG predicting 9‐months ECITT) and cross‐session (9‐months EEG predicting 16‐months ECITT) relationships between brain and behavior. If the residuals were non‐normally distributed, we transformed the independent variable based on the Tucker ladder and re‐ran the models from the first step. As we found differences between sex and brain areas in the goodness of fit of the electrodes, we included sex and goodness of fit in the final model to control for those variables.

## 3 Results

### 3.1 Age‐Related Change and Individual Stability of IC

**FIGURE 3:**
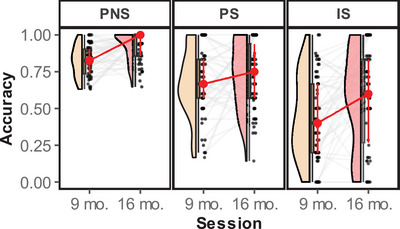
Accuracy development in the ECITT task for each variable: PNS, PS, and IS. Each dot represents a participant, whereas the red and gray lines indicate the average and individual trajectories, respectively. IS = inhibitory switch, PNS = prepotent non‐switch, PS = prepotent switch.

The general accuracy of the ECITT in the direct indexes (i.e., PS, PNS, and IS; marginal *R* ^2^ = 0.32, conditional *R* ^2^ = 0.41; Figure 3 and Table 3) increased between 9‐months and 16‐months sessions (*β* = 0.16, *t* (331.11) = 3.09, *p* = 0.003, 95% CI = [0.06–0.27]). Post‐hoc pairwise comparisons corrected by Holm‐Bonferroni revealed that infants had greater accuracy in the PNS trials than in the IS and PS trials (all zs > 5.82, all *p*s < 0.001). Also, they were more accurate in the PS trials than in the IS trials (*z* = 7.21, *p* < 0.001). Type × Age interaction was not significant (*t* < 2). However, it is possible that some of these trajectories were significant, whereas others may have failed to reach significance. To explore this assumption, we analyzed the development of each trial type. Accuracy increased in the PNS (marginal *R* ^2^ = 0.09, conditional *R* ^2^ = 0.35; *β* = 0.11, *t* (123) = 3.50, *p* < 0.001, 95% CI = [0.05–0.17]) and IS (marginal *R* ^2^ = 0.05, conditional *R* ^2^ = 0.37; *β* = 0.18, *t* (63.52) = 2.77, *p* = 0.007, 95% CI = [0.05–0.30]) but not in the PS trials (marginal *R* ^2^ = 0.02, conditional *R* ^2^ = 0.11; *t* < 2). Regarding the indexes computed, neither the Switching Effect (marginal *R* ^2^ < 0.01, conditional *R* ^2^ = 0.14; *t* < 1) nor the Inhibitory Effect (marginal *R* ^2^ < 0.01, conditional *R* ^2^ = 0.01; *t* = 1) changed between sessions (Figure S4).

**TABLE 3:** Descriptive statistics of the ECITT task.

Session	*N*	Accuracy (% correct)			Inhibition effect	Switching effect
		*IS*	*PNS*	*PS*		
9‐mo.	57	0.44 (0.28)	0.83 (0.11)	0.68 (0.22)	0.24 (0.33)	0.15 (0.17)
16‐mo.	51	0.56 (0.34)	0.91 (0.15)	0.73 (0.23)	0.17 (0.38)	0.18 (0.21)

*Note*: The sample included in the linear mixed model was *n* = 74, considering missing data. The table displays the *M* (SD) accuracy and computed indexes of performance.

Regarding stability, we found that only IS accuracy was related between the two sessions (adj *R* ^2^ = 0.16; *β* = 0.40, *F* (1,73) = 2.74, *p* < 0.01, 95% CI = [0.13 0.80]), with no other significant relationship (all *F* < 1; see Table S3).

### 3.2 Electrophysiological Correlates of EC

**FIGURE 4:**
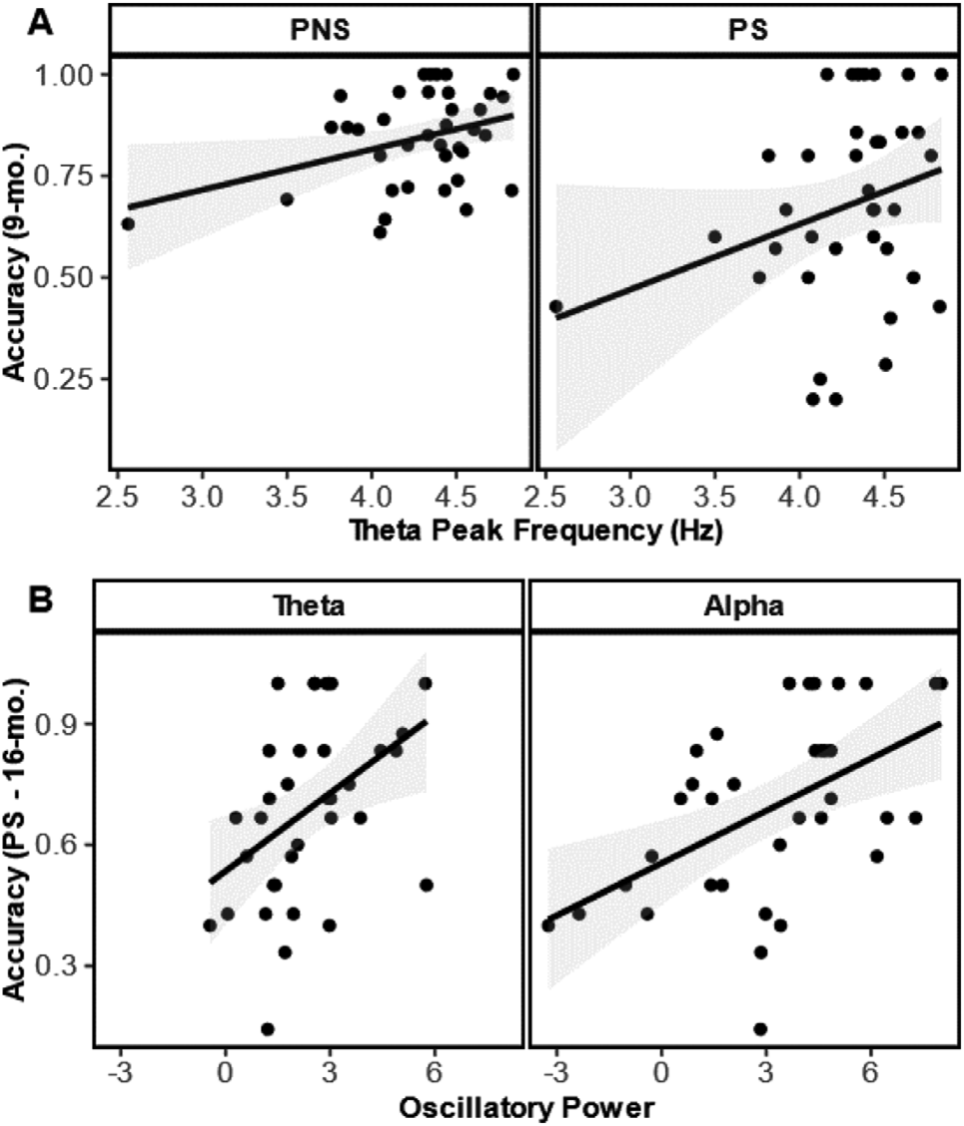
Regression lines of the significant regression models linking behavior and EEG activity concurrently at 9‐mo. (A) and longitudinally from EEG at 9‐mo. to behavior at 16‐mo. (B). EEG = electroencephalography, PNS = prepotent non‐switch, PS = prepotent switch.

Concurrent EEG activity at 9 months was a significant predictor of ECITT performance in the PS (adj. *R* ^2^ = 0.21, *F* (4,31) = 3.286, *p* = 0.023) and PNS (adj. *R* ^2^ = 0.22, *F* (2, 32) = 4.19, *p* = 0.013) trials (Figure 4A), but it was not associated with IS accuracy (*F* < 2). PS accuracy was positively predicted by ITF (*β* = 0.41, *t* (31) = 2.27, *p* = 0.011) and the model included alpha power, but it was not significant (*t* < 2). PNS accuracy was related to ITF (*β* = 0.41, *t* (32) = 2.71, *p* = 0.030).

With respect to concurrent regressions at 16 months, neither the PNS nor the IS accuracy fittest model included any independent variables. In addition, PS at 16 months was not related to concurrent EEG activity (*F* < 1).

In the longitudinal analysis, PS accuracy at 16‐months of age was significantly predicted by the EEG at 9‐months session (adjusted. *R* ^2^ = 0.50, *F* (4, 30) = 9.75, *p* < 0.001; Figure 4B) by alpha (*β* = 0.55, *t* (30) = 4.49, *p* < 0.001) and theta oscillatory power (*β* = 0.42, *t* (30) = 3.43, *p* = 0.002). However, IS and PNS (all *F*s < 2) at the 16‐month session were not significantly predicted by EEG activity at 9 months. See Table 4 for further details on the models. A more detailed description of EEG activity can be found in Figures S5 and S6, and Table S4.

**TABLE 4:** Linear regression models predicting concurrent performance in the ECITT task.

Sessions				Overall model				Regression parameters		
		Predictor		*df*	Adj. *r^2^*	*F*	*B (SE)*	*95%* CI	*β*	*t*
Concurrent	*9‐mo*.	*PNS*	Intercept	3, 32	0.22	4.19^*^	0.48 (0.18)	[0.11–0.85]	—	2.67^*^
			ITF	—	—	—	0.11 (0.04)	[0.03–0.19]	0.41	2.71^*^
		*PS*	Intercept	4, 31	0.21	3.28^*^	0.02 (0.40)	[−0.81 to 0.84]	—	<1
			ITF	—	—	—	0.23 (0.10)	[0.02–0.43]	0.41	2.27^*^
			Alpha Osc.	—	—	—	−0.02 (0.02)	[−0.06 to 0.01]	−0.25	−1.41
		*IS*	Intercept	4, 31	0.06	1.58	−0.93 (0.83)	[−2.66 to 0.81]	—	−1.09
			ITF	—	—	—	0.12 (0.10)	[−0.09 to 0.34]	0.21	1.19
			Exponent	—	—	—	0.53 (0.30)	[−0.09 to 1.14]	0.33	1.74
	*16‐mo*.	*PNS*	Intercept	2, 34	0.00	<1	−0.11 (8.33)	[−17.04 to 16.83]	—	<1
		*PS*	Intercept	3, 33	0.00	1.01	7.25 (19.48)	[−32.40 to 46.88]	—	<1
			IAF	—	—	—	−0.15 (0.09)	[−0.34 to 0.03]	−1.67	−1.67
		*IS*	Intercept	2, 34	0.00	1.06	1.59 (25.20)	[−49.61 to 52.80]	—	<1
Longitudinal		*PNS*	Intercept	2, 34	0.02	<1	0.90 (0.06)	[0.77–1.02]	—	14.77^***^
		*PS*	Intercept	4, 30	0.50	9.725^***^	0.47 (0.12)	[0.23–0.71]	—	4.06^***^
			Theta Osc.	—	—	—	0.06 (0.02)	[0.02–0.10]	0.42	3.43^**^
			Alpha Osc.	—	—	—	0.05 (0.01)	[0.03–0.07]	0.55	4.49^***^
		*IS*	Intercept	3, 31	0.02	1.26	1.25 (0.57)	[0.40–2.72]	—	2.75^*^
			ITF	—	—	—	−0.24 (0.13)	[0.53–0.03]	−0.32	−1.84

*Note*: CI estimates were computed using 5000 bootstraps. All analyses were controlled for sex and goodness of fit of the aperiodic/oscillatory modeling of brain activity.

Abbreviations: IAF = individual alpha frequency, IS = inhibitory switch accuracy, ITF = individual theta frequency, PNS = prepotent non‐switch accuracy; PS = prepotent switch accuracy.

*** *p* < 0.001.

** *p* < 0.01.

* *p* < 0.05.

## 4 Discussion

The main goal of the current research was to study the development of EC from infancy to toddlerhood and examine its relationship with oscillatory/aperiodic brain activity at rest in a longitudinal sample of 9 to 16–18 month‐old babies. We evaluated infants’ EC by means of their performance of the ECITT protocol, which had been previously demonstrated to be appropriate for babies as young as 10 months of age (Fiske et al. 2022; Holmboe et al. 2021; Hendry et al. 2022). Importantly, in the current study, we showed that ECITT is also feasible in 9‐months old infants. Overall, our results indicate that there is a significant development of inhibitory skills in the transition from infancy to toddlerhood, despite a persistent difficulty switching attention from one target location to another on a trial‐by‐trial basis. Moreover, we found that faster theta peak frequency (ITF) significantly predicted performance of PS and PNS trials at 9 months of age, whereas both theta and alpha oscillatory power at 9‐months were significant longitudinal predictors of infants’ CF at 16 months of age.

### 4.1 EC Development

Unlike prior studies using the ECITT (Holmboe et al. 2021; Hendry et al. 2022; Fiske et al. 2022; Lui et al. 2021), we separately analyzed prepotent trials based on whether they were preceded by a change in the target location (PS vs. PNS trials). At both 9 and 16 months of age, participants experienced greater difficulty touching the target button on the prepotent location when it was previously displayed at the non‐prepotent location compared to repeating the prepotent location. This switching cost implies that infants must adjust their attention to the target's location on a trial‐by‐trial basis in order to respond successfully. In fact, infants who were better able to switch between prepotent and inhibitory trials (in both directions) also made fewer errors in the A‐not‐B task in other studies (Hendry et al. 2022). This result demonstrates a significant cost of adjusting the location of attention on a trial‐by‐trial basis in the performance of tasks in which the spatial location is a relevant feature of the target. Additionally, over and above the switching cost, we found that responses to IS trials were the most challenging for infants of both ages. IS trials require withholding a strong response prepotency toward the prepotent location of the target (bear in mind that IS trials were preceded by PNS trials ∼83% of the time), in addition to effective switching of attention to the opposite location. Therefore, ECITT provides a measure of both CF and IC, which can be dissociated in terms of behavioral accuracy.

Age‐related changes in task performance revealed different developmental trajectories for inhibition and switching flexibility. Developmental changes were observed in PNS trials, suggesting that building a prepotent tendency improves with age. We also observed an improvement in the accuracy of IS trials with age, despite the lack of change in the performance of PS trials. This pattern of results suggests that the observed change in IS with age can be attributed exclusively to the enhancement of IC skills, while the response adjustment cost for changing the spatial location of the target remains of a similar magnitude in this protocol. Indeed, we observed similar age‐related changes (better inhibition and accuracy in the non‐switch trials at 16 months) when computing the indices in the ECITT task, as in previous studies (Supporting Results). This pattern reveals an additional dissociation between the flexibility of attention and IC, which is corroborated by its differential association with brain oscillatory activity. However, this differed from previous experiments studying development from 10 to 16 months of life (Hendry et al. 2022), which might be attributed to the wider range of age explored in this study.

Finally, in line with the results of Hendry et al. (2022), we found significant individual stability in the performance of the IS trials. Performance in IS trials at 9 months was positively correlated with the performance of the same trials at 16 months of age. This was significant even after controlling for PS and PNS accuracy, although both significantly contributed to predicting IS performance (Table S5). This result suggests that the building blocks of EC (inhibition and switching) emerge in infancy and are likely to be based on brain mechanisms that, although very likely to be subject to both environmental and constitutional variables, show a certain degree of developmental stability in this early maturational period (Bornstein 2014; Conejero et al. 2023; Hendry et al. 2016).

### 4.2 Brain Oscillatory Activity and EC Development

Although other studies have shown a relationship between frontal activity and aspects of infants’ cognition, our study is the first to dissociate between oscillatory and aperiodic components during EEG recording at rest and to associate this brain activity with performance on the ECITT task. Our results revealed specific concurrent and longitudinal associations between task performance and frontal oscillatory power and ITF; however, we did not find significant relationship with the aperiodic exponent component. This finding highlights the relevance of oscillatory brain activity when predicting high‐order cognitive development, in agreement with prior findings (e.g., Broomell, Savla, Bell 2019; Broomell et al. 2021).

Oscillatory power of the theta band at 9‐months was a positive predictor of the PS trials at 16‐months, and ITF was a concurrent predictor in the PNS and PS trials at 9‐months of age. This is consistent with results from previous studies that found theta to be a predictor of cognitive skills in infancy and childhood/adulthood (Braithwaite et al. 2020; Perone, Palanisamy, and Carlson 2018) and the involvement of ITF in cognitive control (Senoussi et al. 2022). Regarding to oscillatory power, the studies of Jones et al. (2020) and Braithwaite et al. (2020) found better non‐verbal cognitive abilities in infants who showed greater theta power increment in a resting state protocol several months before. In contrast, previous studies had found a negative relationship between theta power and intelligence in adulthood (Tan et al. 2023), and duration of attention and executive function in infancy and early childhood (Perone, Palanisamy, and Carlson 2018; Perone and Gartstein 2019). Thus, the findings regarding theta activity are mixed depending on the approach used to compute power. Studies that found a negative relationship used relative power ratios, while others used power variation. As theta relative power and other derivatives (e.g., theta/beta ratio) are influenced by the aperiodic exponent (Donoghue, Dominguez, and Voytek 2020; Rico‐Picó et al. 2023), a negative relationship between theta and cognitive capacities may occur due to age‐related flattening of the background curve (e.g., Schaworonkow and Voytek 2021; Cellier et al. 2021; Rico‐Picó et al. 2023). Indeed, a steeper background curve (i.e., a larger exponent) has been associated with attention deficit, hyperactivity risk, and poorer executive functions in an autism risk sample of infants (Begum‐Ali et al. 2022; Carter Leno et al. 2022; Karalunas et al. 2021). In the current study, theta oscillatory power was isolated from aperiodic background activity, which may be more similar to the evoked and modulation paradigms. Therefore, our results are consistent with the involvement of theta oscillations as a marker of cognitive control (Cavanagh and Frank 2014; Conejero et al. 2016; Köster et al. 2021).

Regarding alpha oscillatory power, we found a positive longitudinal relationship with PS accuracy. This is consistent with the results of previous studies that found an association between frontal alpha power and performance in the A‐not‐B task (Broomell et al. 2021) and other EF experimental procedures (Hofstee et al. 2022), which makes alpha key to the development of EC (Cuevas and Bell 2022). In fact, alpha has been consistently related to top‐down processes involved in visuospatial attention, cognitive control, and brain communication (Clayton et al. 2018; Fries 2015; Klimesch, Sauseng, and Hanslmayr 2007).

Previous studies have reported early developmental trajectories of alpha and theta oscillatory activity. They have shown a steady increase in both IAF and ITF (e.g., Marshall, Bar‐Haim, and Fox 2002). Although alpha oscillatory power increases with age, theta oscillatory power presents an inverted “*u*” shaped trajectory (Rico‐Picó et al. 2023; Schaworonkow and Voytek 2021). Given these trajectories, our regression models indicated that more mature patterns of brain oscillatory activity at 9 months in alpha and theta rhythms are linked to better EC functioning. This suggests that the presence of such oscillatory activity may be necessary for the emergence of EC at the end of the first year of life. Furthermore, our results indicated that the alpha and theta bands were longitudinal co‐predictors of performance in PS trials in toddlerhood. In PS trials, infants must suppress the tendency to repeat responding on the same side as the previous trial, in addition to disengaging their attentional focus from the previous location and reorienting to the new one. Thus, alpha and theta bands may jointly contribute to infants’ performance in attention switching and behavioral suppression. For instance, a study with adults attributed reactive (vs. proactive) cognitive control to theta (vs. alpha) bands (Clements et al. 2021). This makes it feasible that both bands support differential although contributive roles to infant cognition (see Saby and Marshall 2012; Cuevas and Bell 2022).

In our study, we did not find a significant contribution of the aperiodic exponent to ECITT performance. This is consistent with a recent study that found that children who scored lower on the Behavioral Inhibition Questionnaire had the same exponent values as their peers (Ostlund et al. 2022). However, previous studies have reported the contribution of aperiodic activity to cognition in offline and online paradigms in developmental and adult samples (e.g., Donoghue et al. 2020; Karalunas et al. 2021). For instance, a recent study by Carter Leno et al. (2022) found an interaction between the aperiodic exponent and EC to predict autistic traits. Therefore, despite the lack of a relationship between aperiodic activity and behavior in our study, aperiodic activity may be relevant to the development of particular cognitive skills, and more studies are needed to fully understand its relationship with infant behavior.

Finally, our results revealed longitudinal and concurrent associations between brain oscillatory activity and task performance at 9 months but not at 16 months of age. There is good evidence that rs‐EEG can be a predictor of later behavior (Brito et al. 2016; Jones et al. 2020; Whedon, Perry, and Bell 2020). However, some studies have found concurrent, but not longitudinal, relationships between rs‐EEG and behavior (Leno et al. 2021). Thus, it is important to consider the time points of the measurements when examining the factors that impact the change and stability of the measures. In this sense, introducing trajectories or individual slopes (see Whedon, Perry, and Bell 2020, for example) provides valuable information about how the state of maturation at a particular time point predicts developmental changes in cognitive skills that might reconcile the literature.

### 4.3 Strengths, Limitations, and Future Directions

The current study presents some strengths that are worth mentioning. Firstly, the longitudinal design facilitated the exploration of longitudinal variation and stability of the cognitive skills targeted by the ECITT task. Additionally, it enabled the investigation of both concurrent and longitudinal (EEG at 9‐months, and ECITT at 16‐months sessions) relationships between resting‐state brain activity and individual differences in the performance of the ECITT task. Secondly, the utilization of the ECITT task yielded behavioral EC markers that are less dependent on memory processes compared to previous tasks such as the A‐not‐B (Holmboe et al. 2021). Moreover, the decomposition of EEG activity into aperiodic and oscillatory components provided a more precise measurement of power‐spectrum. Consequently, this approach addressed the potential influence of aperiodic activity on previously reported correlations between alpha/theta power and individual differences in infants' cognitive capacity.

However, some limitations are present in this research. The current study was limited by its relatively modest sample size. This was in part due to the COVID‐19 pandemic, which forced laboratories to interrupt activity for a period of several months. This negatively impacted the attrition rate of families and enforced us to widen the age window for the 16‐month‐old session. However, despite the modest sample size, our findings are in line with those of Hendry et al. (2020), with similar performances observed in prior studies. Furthermore, we did not find any significant correlations between age in days and performance on the ECITT task in the 16–18‐month‐old session (Table S6). Thus, age variability did not appear to affect our results in the 16‐months‐old session.

Although one of the main advantages of ECITT is that it allows the administration of a greater number of trials than other infant‐appropriate EC tasks (e.g., the A‐not‐B task), the ECITT version still presents a limited number of trials. In addition, the probability of occurrence of a non‐switch inhibitory trial is low (∼13%). New variations of ECITT, increasing the number of trials, and including switch and non‐switch inhibitory trials in an equivalent proportion will help to further dissociate the contribution of CF and IC processes in the performance of this task.

Regarding the electrophysiological measurement, we computed the oscillatory power, isolating it from the aperiodic background curve. We based our preprocessing on the assumption that the signal is stationary. However, narrowband activity can appear as a transient burst that may not generate a peak over the aperiodic power‐spectrum curve (Rayson et al. 2022; Zich et al. 2020). Therefore, extracting the properties of transient bursts may benefit the study of other bands that present evoked patterns, such as beta, and dissociate between burst and rhythmic activity. Future studies could potentially investigate brain activity using electroencephalography (EEG) while infants perform the ECITT task. This approach would enable the examination of induced brain activity in aperiodic parameters and theta and alpha bands by directly comparing neural responses of infants' brains during inhibitory versus prepotent trials. Such research would extend the previous findings of Fiske et al. (2022, 2024), which utilized fNIRS to demonstrate the recruitment of frontal brain areas when infants face conditions involving IC.

## 5 Conclusion

We present evidence of the early emergence of EC processes at the end of the first year of life. Our results indicate that, between infancy and toddlerhood, IC skills improve when using a newly developed task (ECITT) that permits a more fine‐grained measurement of EC (i.e., IC and CF) than previously used measures (A‐not‐B task and parent‐reported questionnaires). This is in line with recent research (Hendry et al. 2022) and supports the feasibility of the ECITT with an independent longitudinal sample. In addition, we found that frontal rs‐EEG oscillatory power in the alpha and theta bands were concurrent (ITF at 9 months) and longitudinal predictors of children's EC skills. This contributes to the understanding of the relationship between intrinsic brain function and EC. Other studies have reported a link between brain activity at rest with intelligence (Braithwaite et al. 2020), academic performance (Whedon, Perry, and Bell 2020), and even social adjustment (Caporaso, Boseovski, Marcovitch 2019; Fleming et al. 2020). Thus, investigating both neural and behavioral indicators might help identify potential predictors of children's individual differences from early developmental stages.

## Conflicts of Interest

The authors declare no conflicts of interest.

## Supporting information



Supporting information

## Data Availability

Data is available upon request to the corresponding author (rpicoj@ugr.es) and the senior author of the paper (rorueda@ugr.es).
