# Supplementary material for: Oscillatory But Not Aperiodic Frontal Brain Activity Predicts the Development of Executive Control From Infancy to Toddlerhood
Source: Dev Sci. 2025 Feb 9;28(2):e13613. doi: 10.1111/desc.13613 (PMC11807265; doi:10.1111/desc.13613)
Supplement: Supplementary file 1 — Supporting information [file DESC-28-e13613-s001.docx]

**SUPPLEMENTARY INFORMATION**

**Fig. S1**

*Diagram of the participants included in the analysis and reasons for exclusion.*

**
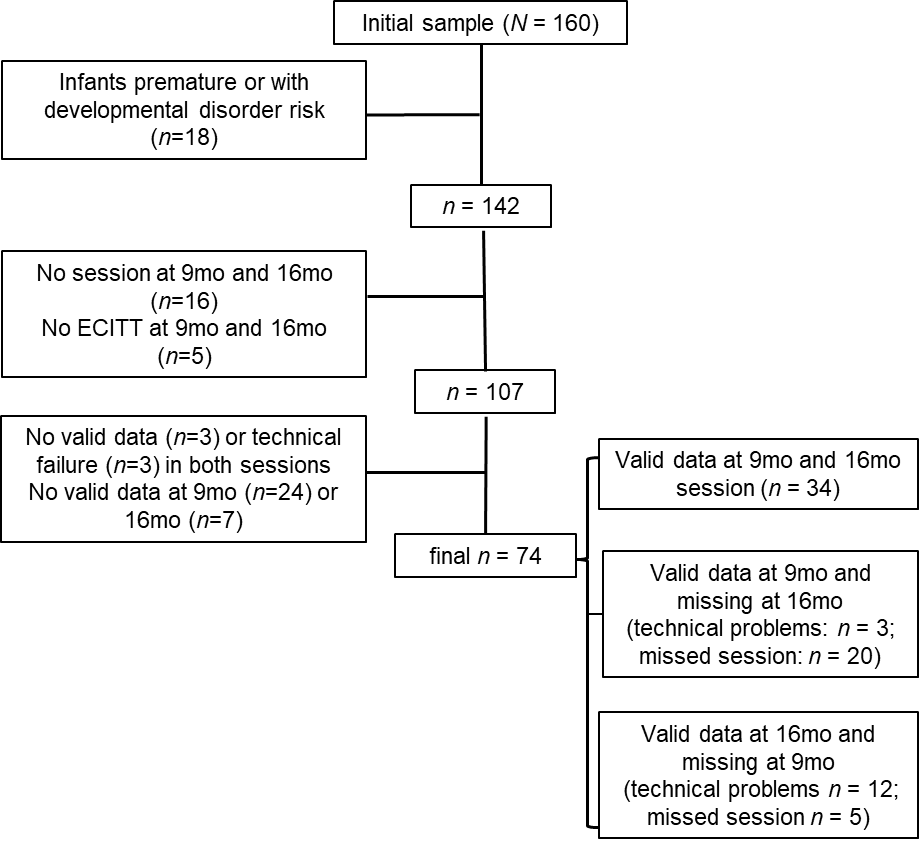
**

**S1. Demographic description of the sample**

Families provided information regarding their socioeconomic information when the babies were 6 months old (mo.). We collected information on education level, occupation level, and the household income corrected. Educational level was coded on a Likert scale ranging from zero (no formal studies) to six (postgraduate studies). Occupation level was coded on a Likert scale from 0 (unemployment) to 9 (high management professions), based on the Spanish National Institute of Statistics (INE) classification of occupations (CNO-11). Finally, we report the income-to-needs ratio. This results from dividing the household annual net income by poverty income, based on the number of family members and year of evaluation. Values below one indicates incomes below the poverty line.

Of the families that answered the questionnaire, most mothers had at least a bachelor’s degree or a higher level of education (*n* = 42), while three, eight, and six mothers had basic education, secondary education, or technical degree, respectively. Regarding parents, the majority had achieved an education level of a bachelor’s degree (*n* = 37), and only one person had no formal studies. In addition, eight fathers had completed elementary school, 16 had completed high school, and seven had finished a technical degree. With respect to occupation, 22 mothers were unemployed and 12 had works related to agriculture or manufacturers. In addition, 34 mothers were employed in works related to administration, teaching, science, and management. Only five fathers were unemployed when babies were 6-mo., while 16 had positions related to agriculture, manufacturers, or basic technicians. The remaining fathers (*n* = 47) held positions in bars, administrations/businesses, professors, scientists, or healthcare facilities. Finally, 22 families were below the poverty line based on the INE data.

**S2. EEG processing**

*S2.1. Relationship between the bubble and the video blocks*

To determine whether bubbles and video blocks were related, we conducted Spearman's correlations corrected by False Discovery Rate (FDR) considering the infants who had sufficient data in both the video and bubble blocks. This analysis was performed separately for each age group and variable in the respective electrode areas included in the regression power (e.g., frontal electrodes in slope and parietal electrodes in alpha peak frequency). To be included in the analysis, children had to have a minimum of five epochs per condition (25s). A total of 36 children (bubbles: *M* = 11.95, *SD* = 5.38, video: *M* = 8.76, *SD* = 4.39) and 29 children (bubbles: *M* = 13.27, *SD* = 9.85, video: *M* = 12.41, *SD* = 7.02) were included in the analysis at 9 and 16 months of age, respectively. This analysis revealed that, independent of age and variable, all measurements were correlated between blocks with values ranging from r = .42 to r = .63 (all *p*s < .05 corrected) in the oscillatory parameters, and r = .67 to r = .77 (all *p*s < .001) in the aperiodic parameters.

| EEG Component | Variable | Session | |
| --- | --- | --- | --- |
|  |  | 9-mo. | 16-mo. |
| Aperiodic | Slope | 0.7*** [0.64 - 0.76] | 0.73*** [0.66 - 0.78] |
|  | Offset | 0.67*** [0.70 - 0.80] | 0.77*** [0.71 - 0.82] |
| Theta Oscillatory | Power | 0.47** [0.37 - 0.55] | 0.42* [0.31 - 0.52] |
|  | Peak Frequency | 0.52** [0.43 - 0.60] | 0.55** [0.45 - 0.64] |
| Alpha Oscillatory | Power | 0.42* [0.32 - 0.51] | 0.63*** [0.54 - 0.70] |
|  | Peak Frequency | 0.52** [0.43 - 0.60] | 0.5** [0.40 - 0.60] |

**Table S1.**

*Spearman´s rho correlation values between bubbles and video trials in the resting-state protocol.*

Note. Confidence intervals were calculated using bootstrapping (*n* = 1000). Asterisks represent the *p* values after FDR correction. *** *p <* .001, ** *p <* .01 **p <* .05

***S.2.2. Spearman-Brown split half reliability***

We assessed the reliability of the oscillatory and aperiodic parameters in our sample to determine the minimum number of epoch required to be included in the analysis. To this aim, we reprocessed the EEG signal epoch by epoch, fitted the aperiodic curve, and extracted the oscillatory parameters individually. An epoch was included if 80% of the electrodes of interest had a fit of R^2^ > .949. Based on these results, we conducted Spearman-Brown split-half correlation in each session for all variables. We tested reliability values from 1 to 20 in 1-epoch increments in 5000 iterations to avoid trial selection bias. This was computed considering the region of interest of each variable. That is, parietal electrodes for alpha and theta peak frequency, and frontal electrodes for oscillatory power and aperiodic parameters. Spearman-Brown results revealed that alpha band and the aperiodic parameters exhibited higher reliability than the theta band (Fig. S2). However, the reliability of the variables reached excellent levels within five epochs (i.e., 25 s of data). With that amount of data, almost all the variables attained values of r_sb_ > .80 (excellent reliability), except the theta peak frequency in both sessions (9-mo. r_sb_ = .76, 9-mo. r_sb_ = .72) and alpha peak frequency at 16-mo. sessions (r_sb_ = .77) that displayed good reliability.


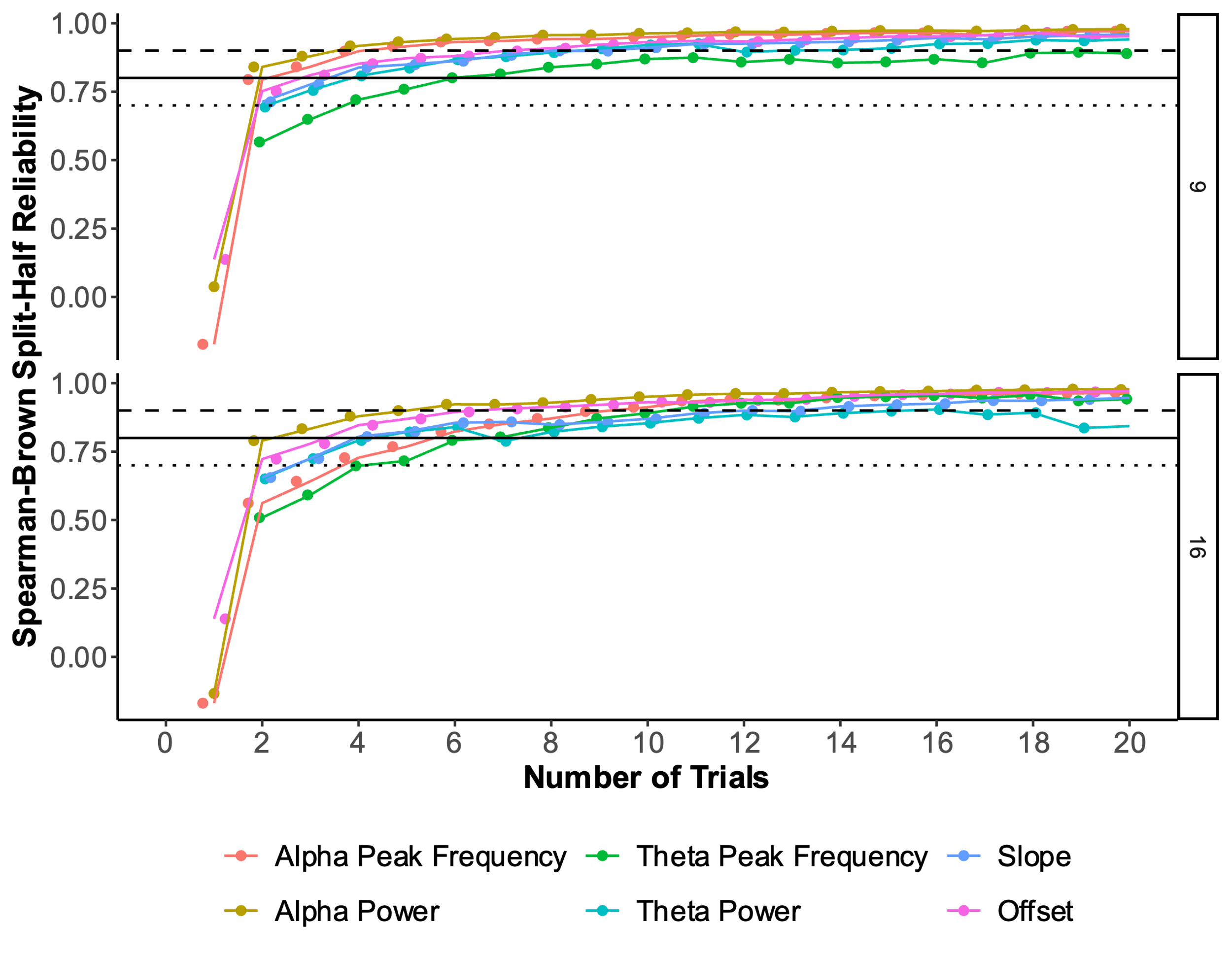
**Fig S2.**

*Spearman-Brown split-half reliability. The figure shows the average value after iterations for all variables divided by age.*

**Table S2.**

*Descriptive statistics of the goodness of fit (R^2^) and percentage of electrodes included in the analysis were divided by sex, analysis type, and ROI. Descriptives are presented as M (SD).*

| **Sessions** | | **Sex** | ***R*^2^** | | | **Electrodes Included %** | | |
| --- | --- | --- | --- | --- | --- | --- | --- | --- |
|  |  |  | ***Frontal*** | ***Occipital*** | ***Parietal*** | ***Frontal*** | ***Occipital*** | ***Parietal*** |
| **Concurrent** | *9-mo.* | *F* | 99.11 (0.93) | 99.71 (0.25) | 99.58 (0.58) | 0.97 (0.18) | 1 (0) | 0.99 (0.07) |
|  |  | *M* | 98.55 (1.19) | 99.62 (0.36) | 99.39 (0.75) | 0.88 (0.33) | 1 (0) | 0.98 (0.13) |
|  | *16-mo.* | *F* | 99.02 (1.03) | 99.66 (0.31) | 99.59 (0.43) | 0.98 (0.14) | 1 (0) | 1 (0) |
|  |  | *M* | 99.11 (0.96) | 99.71 (0.2) | 99.47 (0.67) | 0.98 (0.14) | 1 (0) | 1 (0) |
| **Longitudinal** |  | *F* | 98.87 (1.08) | 99.63 (0.34) | 99.41 (0.72) | 0.91 (0.28) | 1 (0) | 0.98 (0.14) |
|  |  | *M* | 98.76 (1.1) | 99.67 (0.29) | 99.42 (0.72) | 0.88 (0.32) | 1 (0) | 0.98 (0.13) |

Note. Because the values of *R^2^* were close to 1, we multiplied its value by 100 to obtain more detailed information. F = Female, M = Male.

**
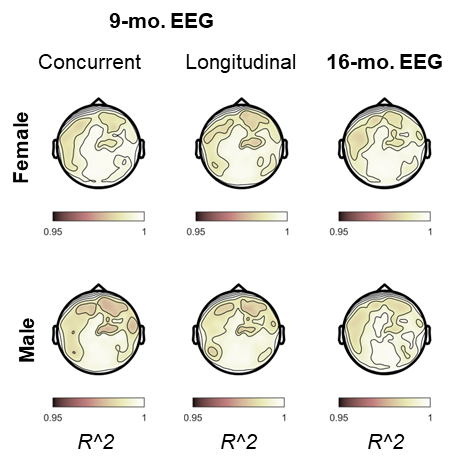
 Fig. S3.**

*Topographical representation of the goodness of fit for each analysis relating EEG to behavior.*

The graphic displays only the participants included as well as the filtered channels incorporated in the average.


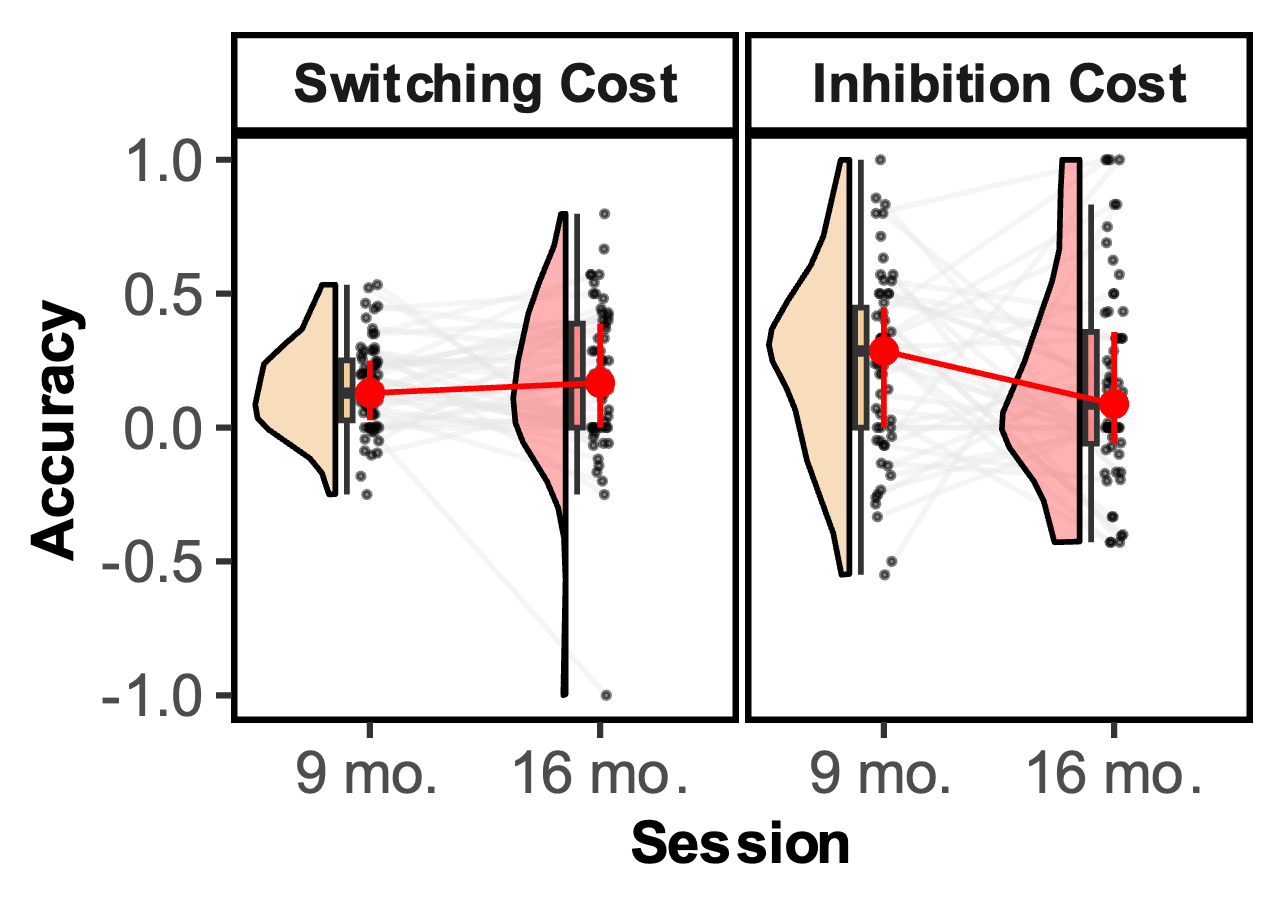


**Fig. S4**

*Development of switching and inhibition cost indices.*

Each dot represents a participant, and the red and gray lines indicate the average and individual trajectories, respectively.

**Table S3.**

*Linear regression models predicting ECITT performance at 16-mo. based on 9-mo. results.*

|  | **Overall Model** | | | **Regression Parameters** | | | |
| --- | --- | --- | --- | --- | --- | --- | --- |
| **Variable** | *df* | *r^2^* | *F* | *B (SE)* | *95% CI* | *β* | *z* |
| PNS | 1, 72 | 0.03 | <1 | -0.28 (0.25) | [-0.78 0.21] | -0.18 | -1.12 |
| PS | 1, 72 | 0.01 | <1 | 0.09 (0.16) | [-0.21 0.41] | 0.10 | 0.62 |
| **IS** | **1, 72** | **0.16** | **7.72**** | **0.47 (0.17)** | **[0.13 0.80]** | **0.40** | **2.74**** |
| SE | 1, 72 | 0.02 | <1 | 0.09 (0.16) | [-0.21 0.41] | 0.10 | 0.62 |
| IE | 1, 72 | <0.01 | <1 | 0.06 (0.19) | [-0.32 0.43] | 0.10 | 0.29 |

Note. The regression model included FIML to account for missing data (*N* = 74). Beta and CI estimates were computed using 5000 bootstraps. PNS = Prepotent Non-Switch accuracy; PS = Prepotent Switch accuracy; IS = Inhibitory Switch accuracy; SE = Switching Effect index; IE = Inhibitory Effect index. ** *p* < .01.

**Fig. S5.**

*Oscillatory power and aperiodic exponent were extracted from the frontal clusters of the infants included in the analysis.*


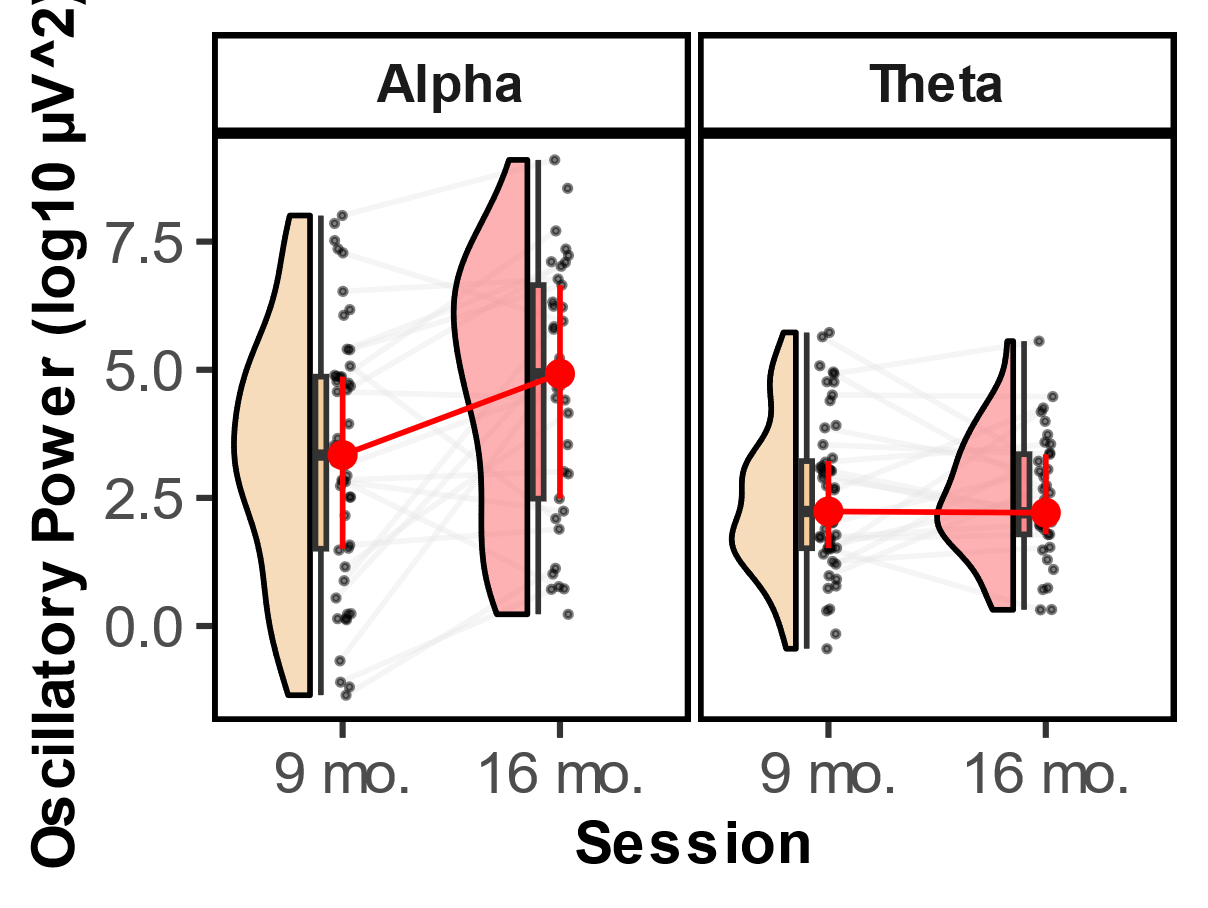

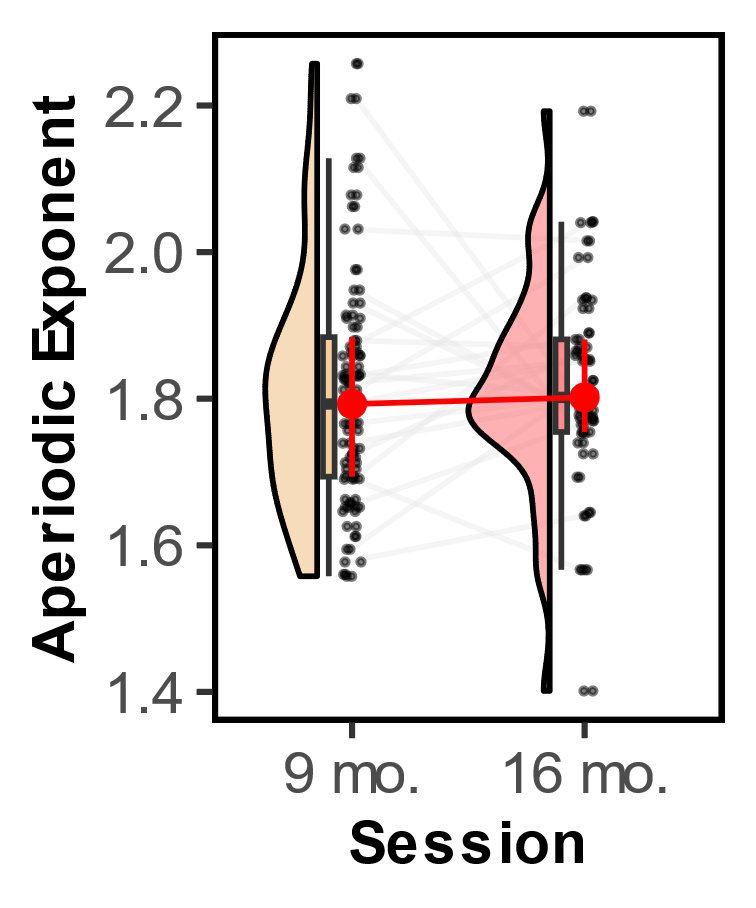


Note. Each dot represents a participant, whereas the red and gray lines indicate the average and individual trajectories of the participants in both sessions.


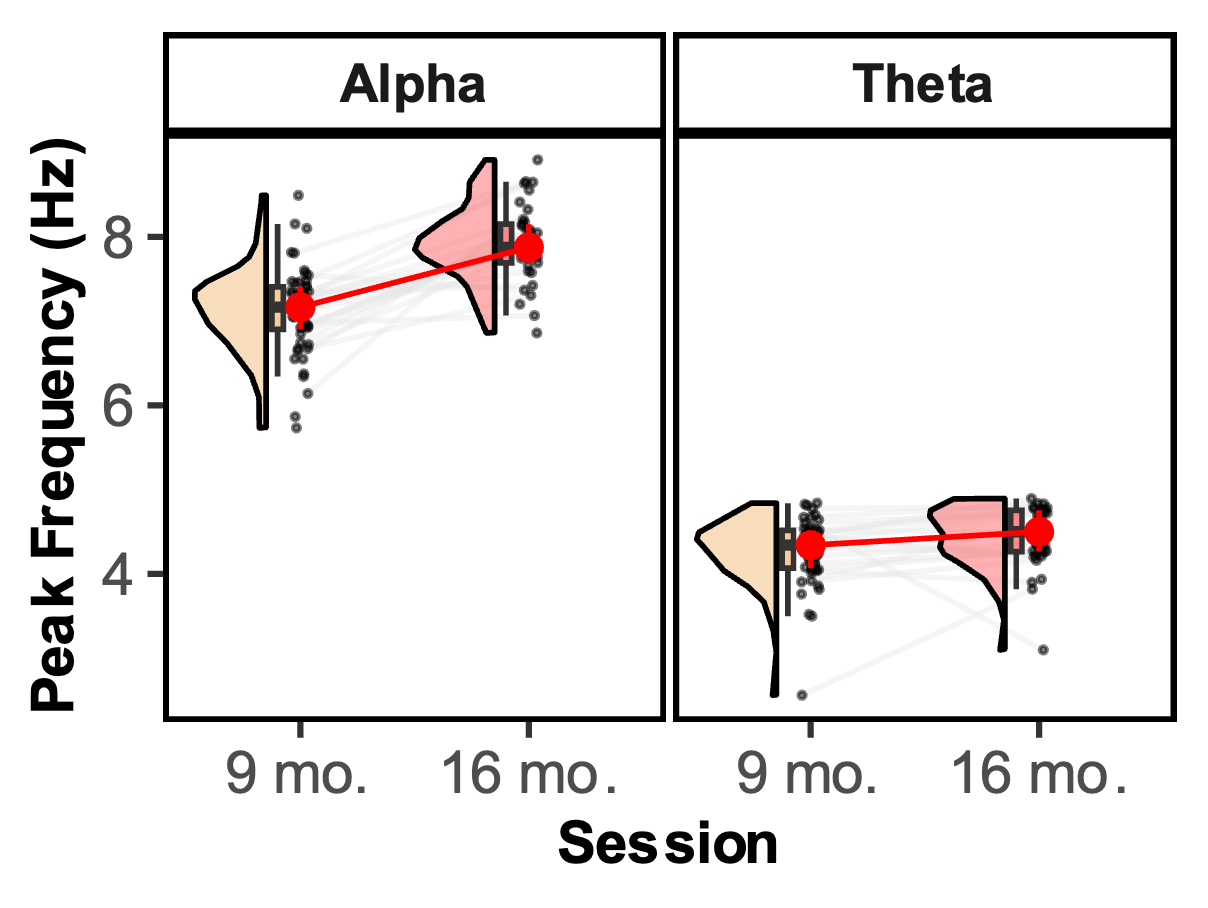
**Fig. S6**

*Peak frequency in the theta and alpha bands extracted from the parieto-occipital cluster in the infants was included in the analysis.*

Each dot represents a participant, whereas the red, gray lines indicate the average and individual trajectories of the participants included in both sessions.

**Table S4.**

*Descriptive statistics of the oscillatory and aperiodic parameters included in the regression models. The table displays the M (SD) of the oscillatory power, peak frequency, and aperiodic exponent divided by the session.*

| **Session** | **N** | **Oscillatory Power** | | **Peak Frequency (Hz)** | | **Aperiodic Exponent** |
| --- | --- | --- | --- | --- | --- | --- |
|  |  | *Theta* | *Alpha* | *Theta* | *Alpha* |  |
| **9-mo.** | 52 | 2.58  (1.72) | 3.48  (2.82) | 4.30  (0.48) | 7.09  (0.79) | 1.82  (0.26) |
| **16-mo.** | 37 | 2.50  (1.44) | 4.67  (2.87) | 4.41  (0.42) | 7.92  (0.63) | 1.81  (0.23) |

Note. The participants in the table are all the infants who were included in the three regressions conducted.

**Table S5.**

*Linear regression models predicting Inhibitory Switch performance based on all performance indexes at 9 months session.*

|  | **Overall Model** | | | **Regression Parameters** | | | |
| --- | --- | --- | --- | --- | --- | --- | --- |
| **Variable** | *df* | *r^2^* | *F* | *B (SE)* | *95% CI* | *β* | *z* |
| Model | 3, 68 | .09 | 10.97** |  |  |  |  |
| **PNS** |  |  |  | **0.94 (0.27)** | **[0.41 1.43]** | **.61** | **3.49***** |
| **PS** |  |  |  | **-1.59 (0.59)** | **[-2.74 -.43]** | **-.48** | **-2.70**** |
| **IS** |  |  |  | **0.41 (0.16)** | **[0.09 0.72]** | **.33** | **2.55*** |

Note. The regression model included FIML to account for missing data (*N* = 74). Beta and CI estimates were computed using 5000 bootstraps. PNS = Prepotent Non-Switch accuracy; PS = Prepotent Switch accuracy; IS = Inhibitory Switch accuracy; SE = Switching Effect index; IE = Inhibitory Effect index. *** *p <* .001, ** *p <* .01 **p <* .05

**Table S6.**

*Correlations (Spearman rho) between session age (in days) and performance on the ECITT task.*

|  | **PNS** | **PS** | **IS** | **SE** | **IE** |
| --- | --- | --- | --- | --- | --- |
| **9-mo** | .004  [-.25 .26] | -.03  [-.30 .22] | -.09  [-.33 .16] | .03  [-.23 .20] | .06  [-.18 .28] |
| **16-mo** | .07  [-.21 .31] | .14  [-.12 .37] | .06  [-.19 .31] | -.10  [-.34 .15] | .04  [-.24 .30] |

Note. The correlations were within age and included all infants with valid data at that age. The sample sizes were 60 (32 male) at 9 months and 60 (29 men) at 16 months. Confidence intervals were extracted from 5000 bootstrap replicates. *PNS* = Prepotent Non-Switch accuracy; *PS* = Prepotent Switch accuracy; *IS* = Inhibitory Switch accuracy; *SE = Switching Effect index; IE =* Inhibitory Effect index.

**SUPPLEMENTARY RESULTS**

To determine whether the results employed in this study had trajectories different from those previously reported (e.g., Hendry et al., 2022), we re-analyzed the data computing the original behavioral indices. The indices were the accuracy in the prepotent and inhibitory trials, without differencing the switching and non-switching positions. In addition, we compared the accuracy between the switching and non-switching trials, regardless of whether the location of the preceding trial. Finally, we evaluated the Inhibitory Index, which was computed by subtracting the inhibitory trial hits from the prepotent trial hits and then inverting the punctuation.

**SR1. Development and stability**

The analysis plan was identical to that used in the main text. In other words, a linear mixed model estimating missing data with the Type of Trial × time to estimate the development, and a linear mixed model regression between the performance to compute the stability of the measures of the ECITT. Descriptions of the performance and stability can be found in SR Tables 1 and 2, respectively. In addition, refer to SR in Figs. 1 and 2.

With regard to the development of inhibitory vs. prepotent trial accuracy (marginal *R*^2^ = .35; conditional *R*^2^ = .46), prepotent trials were more accurate than inhibitory trials in general (*β* = 0.32, *t*(211) = 8.73, *p* < .001, 95% CI = [0.25 – 0.40]) without a significant main effect of Time (*t* < 2) or an interaction effect (*t* < 1). However, when we differentiated between prepotent and inhibitory trials, we found no increment in the prepotent trials (marginal *R*^2^ = .03; conditional *R*^2^ = .22; *t* < 2) but a positive increase in inhibitory trials accuracy (marginal *R*^2^ = .43; conditional *R*^2^ = .49; *β* = 0.15, *t* (104) = 2, *p* = .048, 95% CI = [0.01 – 0.30]).

Regarding switch accuracy (marginal *R*^2^ = .45; conditional *R*^2^ = .55), the trials that required location change had worse performance (*β* = -0.27, *t*(211) = -9.97, *p* < .001, 95% CI = [-0.32 – -0.22]), and the older the children, the better their performance (*β* = 0.11, *t*(211) = 2.18, *p* = .030, 95% CI = [0.01 – 0.20]) without a significant interaction (*t* < 1). Nevertheless, when we exploratorily tested the development separately, the non-switch trials increased the accuracy between sessions (marginal *R*^2^ = .07; conditional *R*^2^ = .12; *β* = 0.10, *t* (105) = 2.94, *p* = .004, 95% CI = [0.03 – 0.17]), but it was not significant in the switch trials (*t* < 2). Finally, the inhibitory cost index did not vary over time (*t* < 1).

The stability between sessions was computed only in terms of accuracy in switch, non-switch, and inhibitory trials, plus the inhibitory cost index, as the accuracy in the prepotent trial model did not converge. Accuracy in the switch (adj *R*^2^ = .16; *β* = 0.52, *F* (1,73) = 3.78, *p* < .001, 95% CI = [0.28 – 0.88]) and inhibitory trials (adj *R*^2^ = .16; *β* = 0.49, *F* (1,73) = 3.49, *p* < .001, 95% CI = [0.23 – 0.83]) at 9-mo. were a significant predictor of 16-mo. performance. This was not observed for other variables (*F*s < 1).

**Table SR1.**

*Descriptive statistics of the ECITT task. The table displays the mean (standard deviation) for direct accuracy and the computed indexes.*

| **Session** | **Accuracy** | | | | **IC** |
| --- | --- | --- | --- | --- | --- |
|  | *Prpt.* | *Inhb.* | *Switch* | *Non-Switch* |  |
| **9-mo.** | 0.83 (0.11) | 0.50 (0.28) | 0.59 (0.19) | 0.86 (0.11) | -0.29 (0.42) |
| **16-mo.** | 0.87 (0.11) | 0.56 (0.30) | 0.62 (0.22) | 0.92 (0.10) | -0.30 (0.33) |

The sample size included in the linear mixed model was the same as in the main text: 74, considering the missing data.

**Table SR2.**

*Linear regression models predicting the ECITT performance at 16mo based on 9mo results.*

|  | **Overall Model** | | | **Regression Parameters** | | | |
| --- | --- | --- | --- | --- | --- | --- | --- |
| **Variable** | *df* | *r^2^* | *F* | *B (SE)* | *95% CI* | *β* | *z* |
| **Inhb**. | **1, 73** | **0.24** | **9.02**** | **0.53 (0.15)** | **[0.23 0.83]** | **0.48** | **3.49***** |
| **Switch** | **1, 73** | **0.27** | **9.87**** | **0.58 (0.15)** | **[0.28 0.88]** | **0.52** | **3.78**** |
| Non-Switch | 1, 73 | <0.01 | <1 | 0.04 (0.15) | [-0.25 0.34] | 0.05 | 0.27 |
| IC | 1, 73 | <0.01 | <1 | -0.02 (0.13) | [-0.29 0.24] | -0.03 | -0.16 |

Note. The regression model included FIML to account for missing data (*N* = 74). Inhb. = inhibitory trial accuracy, switch = switching trial accuracy, non-switch = non-switch trial accuracy, IC = inhibitory cost. Beta and CI estimates were computed using 5000 bootstraps. *** *p* < .001. ** *p* < .01.

**Fig. SR1**

*Development of a prepotent (Prpt.), and inhibitory (Inhb.) accuracy (left), and age-related changes in the switch vs. no-switch trials (right).*


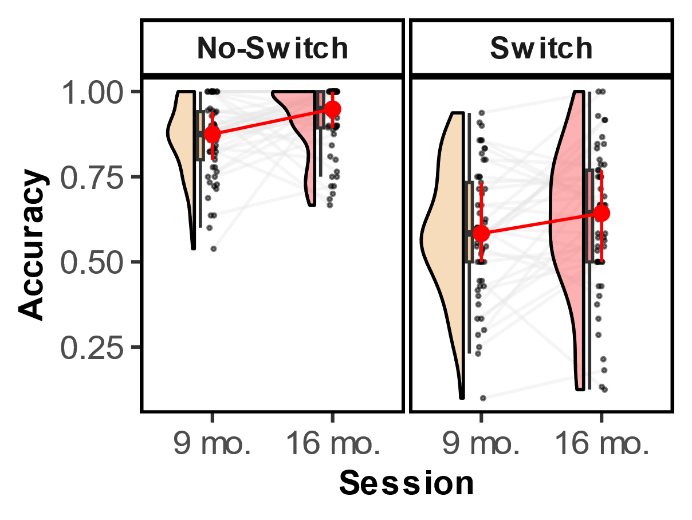

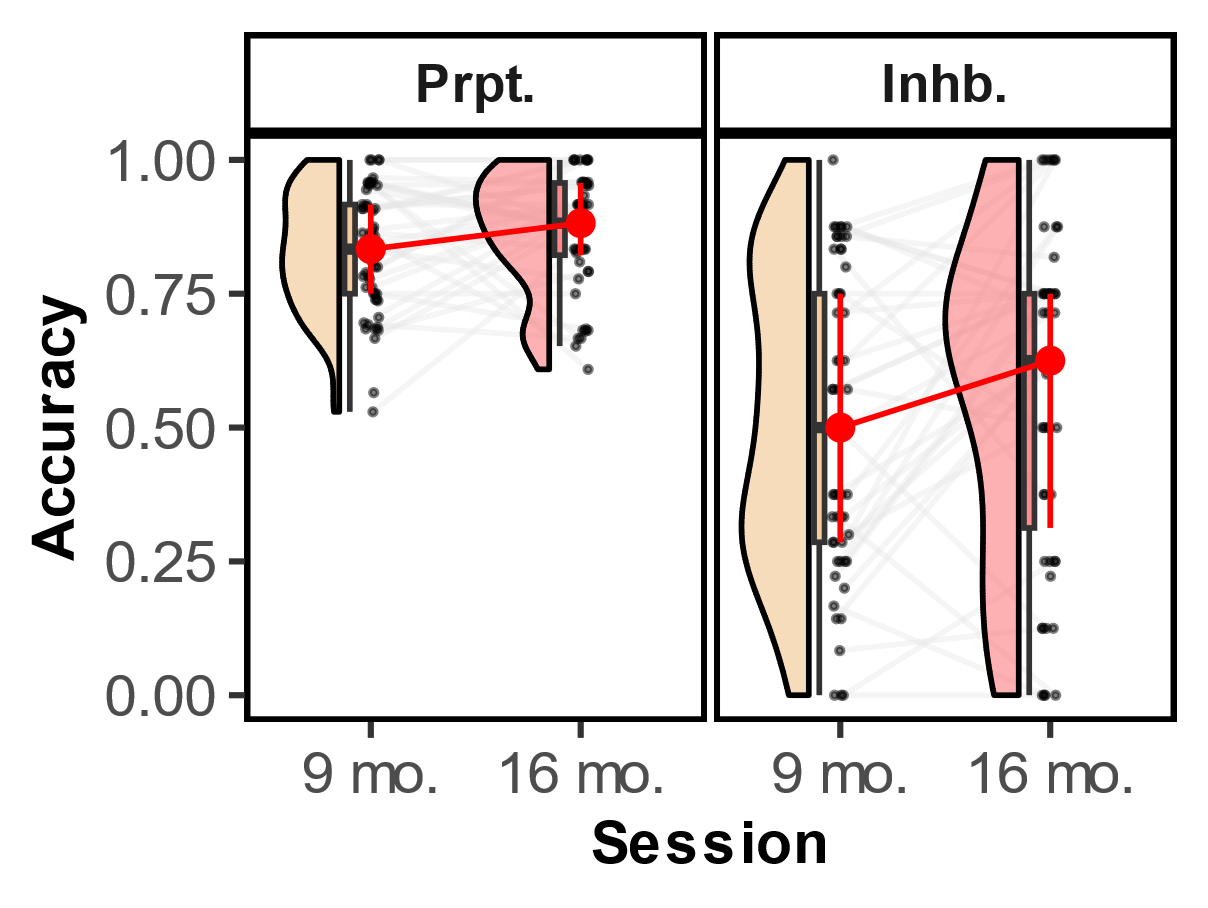


Each dot represents a participant, whereas the red, gray lines indicate the average and individual trajectories, respectively.


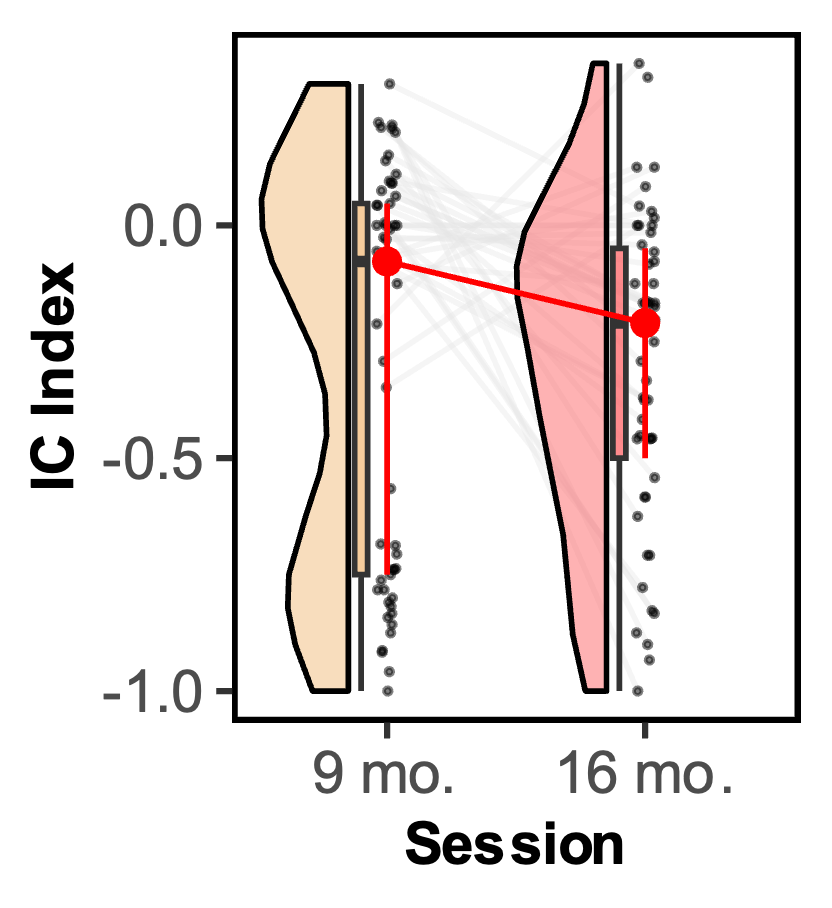
**Fig. SR2**

*Development of prepotent Inhibitory Cost (IC) index.*

Each dot represents a participant, whereas the red, gray lines indicate the average and individual trajectories, respectively.
